# Proceedings of the DeWitt County Medical Society

**Published:** 1860-12

**Authors:** C. Goodbrake

**Affiliations:** Secretary


### PROCEEDINGS OF THE DE WITT COUNTY MEDICAL SOCIETY.

—

REPORTED BY C. GOODBRAKE, M. D., SECRETARY.

—

The Society met in semi-annual session, at the office of Drs. Wright and Davis, in Wapella, on Tuesday the 2d day of October, 1860. Dr. John Wright in the chair.

The minutes of the previous meeting were read and adopted.

Dr. Thomas W. Davis, who had previously passed the Board of Censors, was, on motion of Dr. Goodbrake, duly elected a member of the society.

Dr. Wright reported a case of wounded knee-joint. The patient, a man about forty-five years of age, had received a small wound with a knife, such as is used for cutting up corn, immediately below the patella on the inner side of the knee. The joint swelled very rapidly and became extremely painful. The case was treated with warm fomentations to the knee; quinine, opium and calomel were administered internally. The case was progressing to a favorable result.

Dr. Davis reported three cases of typhoid fever that came under his care, which presented some singular features. The patients, in the three cases, had all the usual symptoms of this disease: such as head-ache, lassitude, want of appetite, tongue coated with a whitish fur, very narrowly edged with red, bowels more or less tender, with a disposition to diarrhea, pulse from 95 to 110 per minute. These symptoms lasted two or three weeks; the patients all this time being able to go about the house, and what was remarkable, they became as emaciated during the period of their sickness, as others whom the doctor attended at the same time, and who became very low, so much so, as not to be able to turn themselves in the bed. The doctor's treatment consisted in tonics, animal broths, with opium and nitrate of silver, one quarter grain of each, every six hours, when considered necessary.

Dr. Goodbrake reported two cases of puerperal convulsions; both were primapara cases. The first was that of a woman,

30 years of age, the convulsions set in about the time the os tincae began to dilate. She was bled freely, chloroform was administered to control as much as possible, the convulsions. She was delivered by the aid of the forceps, of a living child, nine hours after the accession of the first convulsion. The woman died twenty-four hours after the delivery, in a comatose state.

The second case was that of a Mrs. I——, aged 17 years, of small stature, short neck, florid countenance, sanguine temperament. This patient was *not bled*; but chloroform was administered to control the convulsions. Ungt. belladonna was applied very freely to the mouth of the womb. She was delivered by the aid of instruments six hours from the time she had the first convulsion. The patient made a good recovery. The Dr. gave it as his experience, that a large percentage of puerperal convulsions occurred in primapara cases; also, that they took place about the time the os tincae commenced dilating, which was usually found rigid and unyielding, and was of the opinion that in all such cases the early and free application of belladonna might prevent these frightful convulsions. He said, that he believed he was warranted in saying, that in several cases where this horrible disease was threatening, he averted it by this treatment.

Cholera Infantum, the regular subject for discussion, was then taken up, when most of the gentlemen present expressed their views on the pathology and treatment of the disease.

Drs. W. W. Adams and T. K. Edmiston were appointed essayists.

On motion, the thanks of the Society were tendered to Drs. Wright and Davis and their ladies for the sumptuous dinner served up for the members at this session.

Typhoid Fever was chosen as the subject for discussion at the next meeting.

The Society then adjourned to meet in quarterly session at Marion on the first Tuesday in January, 1861.

